# Leptin regulates exon-specific transcription of the *Bdnf* gene via epigenetic modifications mediated by an AKT/p300 HAT cascade

**DOI:** 10.1038/s41380-020-00922-0

**Published:** 2020-10-26

**Authors:** Chen Li, Fantao Meng, Yun Lei, Jing Liu, Jing Liu, Jingyan Zhang, Fang Liu, Cuilan Liu, Ming Guo, Xin-Yun Lu

**Affiliations:** 1 Institute for Metabolic & Neuropsychiatric Disorders, Binzhou Medical University Hospital, Shandong, China; 2 Department of Neuroscience & Regenerative Medicine, Medical College of Georgia at Augusta University, Augusta, GA USA; 3 Department of Pharmacology, University of Texas Health Science Center at San Antonio, San Antonio, TX USA

**Keywords:** Neuroscience, Depression, Genetics

## Abstract

Leptin is an adipocyte-derived hormone with pleiotropic functions affecting appetite and mood. While leptin’s role in the regulation of appetite has been extensively studied in hypothalamic neurons, its function in the hippocampus, where it regulates mood-related behaviors, is poorly understood. Here, we show that the leptin receptor (LepRb) colocalizes with brain-derived neurotrophic factor (BDNF), a key player in the pathophysiology of major depression and the action of antidepressants, in the dentate gyrus of the hippocampus. Leptin treatment increases, whereas deficiency of leptin or leptin receptors decreases, total *Bdnf* mRNA levels, with distinct expression profiles of specific exons, in the hippocampus. Epigenetic analyses reveal that histone modifications, but not DNA methylation, underlie exon-specific transcription of the *Bdnf* gene induced by leptin. This is mediated by stimulation of AKT signaling, which in turn activates histone acetyltransferase p300 (p300 HAT), leading to changes in histone H3 acetylation and methylation at specific *Bdnf* promoters. Furthermore, deletion of *Bdnf* in the dentate gyrus, or specifically in LepRb-expressing neurons, abolishes the antidepressant-like effects of leptin. These findings indicate that leptin, acting via an AKT-p300 HAT epigenetic cascade, induces exon-specific *Bdnf* expression, which in turn is indispensable for leptin-induced antidepressant-like effects.

## Introduction

Leptin is produced and secreted from adipocytes [1], circulates in the blood [2], and is transported across the blood–brain barrier [3]. Leptin’s target neurons are distributed widely throughout the brain [4–6], through which leptin exerts pleiotropic effects by activating the signaling form of the leptin receptor (LepRb) [7–9]. While extensive studies have focused on leptin’s actions in the hypothalamus in the regulation of feeding and energy metabolism [10, 11], LepRb is also highly expressed in extra-hypothalamic brain regions including the hippocampus, which is implicated in mood regulation [4, 12–14]. We and others have shown that circulating leptin levels are reduced in chronic stress animal models of depression [15–17], whereas systemic and intracerebroventricular injections of leptin produce antidepressant-like behavioral effects [15, 18–20]. In humans, leptin levels were found to correlate negatively with the severity of depression symptoms [21], Multiple lines of evidence suggest that leptin targets hippocampal neurons to regulate depression-related behaviors. First, direct infusion of leptin into the dentate gyrus of the hippocampus induces an antidepressant-like effect, similar to the effect observed after systemic injection [15]. Second, deletion of LepRb in the dentate gyrus results in depression-like behaviors and attenuates leptin’s antidepressant-like effects [13, 22]. Third, blockade of leptin signaling in the dentate gyrus attenuates the antidepressant-like effects of leptin [14]. These findings support an important role of the hippocampus in mediating leptin’s actions on mood-related behavior. However, the precise molecular mechanisms remain unknown.

Brain-derived neurotrophic factor (BDNF) is a neurotrophin that has been implicated in the pathophysiology of depression and the mechanisms of action of antidepressant drugs [23]. Hippocampal mRNA or protein levels of BDNF are increased by treatment with different classes of antidepressants [24, 25] in rodents and in depressed patients [26]. Antidepressant drugs activate the BDNF receptor, tropomycin receptor kinase B (TrkB), in the hippocampus, suggesting enhanced BDNF release [27]. Furthermore, infusion of BDNF directly into the hippocampus is sufficient to induce antidepressant-like effects [28, 29]. Importantly, the antidepressant-like response to classical antidepressants is attenuated in transgenic mice with disrupted BDNF signaling [27]. In addition, the rapid onset antidepressant effects of ketamine require expression of BDNF [30, 31]. Together, these data indicate that increased BDNF expression and signaling are both necessary and sufficient to produce the behavioral effects of antidepressants.

The *Bdnf* gene in rodents contains eight noncoding 5′ exons (I–VIII), which are alternatively spliced to the 3′ protein-encoding exon IX [32, 33]. Each exon is driven by its own unique promoter sequence for the temporal and spatial specificity of *Bdnf* expression in an activity-dependent manner [34, 35]. All *Bdnf* mRNA isoforms are translated into the same BDNF protein [36]. Specific exon-containing *Bdnf* mRNAs are differentially regulated by stimuli such as changes in neuronal activity [37–39], stress [40–43], and antidepressant treatment [44–47]. However, whether leptin regulates the transcription of the *Bdnf* gene in the hippocampus is unknown.

In this study, we investigated (1) whether, and to what extent, LepRb is colocalized with BDNF in the hippocampus; (2) whether and how leptin regulates exon-specific *Bdnf* expression; (3) whether leptin and deficiencies of leptin or its receptor have opposite effects; and (4) whether *Bdnf* expression in the hippocampus is required for leptin’s antidepressant-like behavioral effects. To address these questions, we employed a genetic approach to investigate colocalization of LepRb and BDNF expression in the brain. Furthermore, we examined exon-specific *Bdnf* expression in response to leptin treatment and impaired leptin signaling and further explored the epigenetic mechanisms underlying leptin-induced regulation of *Bdnf* gene expression. Finally, we determined the effects of loss of BDNF in the hippocampus on behavioral responses to leptin.

## Materials and methods

### Animals

Wild-type C57BL/6J mice (Stock No. 000664), LepRb-ires-Cre mice (Stock No. 008320), Ai14-tdTomato mice (Stock No. 007914), *Bdnf*^*flox/flox*^ (Stock No. 004339), *Bdnf*^*klox/klox*^ mice (Stock No. 021055), and Emx1-Cre mice (Stock No. 005628) were purchased from Jackson Laboratory (Bar Harbor, ME, USA) and maintained as breeding colonies. The *ob/ob* and *db/db* mice and their littermates were obtained by intercrossing *ob/+* or *db/+* mice (*ob/+*, Stock No. 000632; *db/+*, Stock No. 000642; Jackson Laboratory). LepRb-ires-Cre mice were generated with an IRES-NLS-Cre cassette “knocked in” to the region immediately 3′ to the LepRb stop codon [48] to drive Cre expression in all Lepr-expressing cells. Ai14 mice have a loxP-flanked STOP cassette preventing transcription of a CAG promoter-driven tdTomato protein in all cells. Ai14 mice express robust tdTomato fluorescence following Cre-mediated recombination [49]. *Bdnf*^*flox/flox*^ mice possess *loxP* sites on either side of the *Bdnf* coding region [50]. *Bdnf*^*klox/klox*^ mice possess loxP sites flanking the *Bdnf* encoding exon 9, with a polyadenylation sequence upstream of the 3′ *loxP* site, and *lacZ* downstream of the 3′ *loxP* site [51]. All mice were maintained in the C57BL/6J background. Mice were housed in a group of 5 and maintained on a 12 h light/12 h dark cycle with ad libitum access to food and water. The animal protocols used in this study were approved by the Institutional Animal Care and Use Committees of the University of Texas Health Science Center at San Antonio, Binzhou Medical University Hospital, and Augusta University.

To label LepRb neurons, LepRb-ires-Cre mice were crossed with the reporter Ai14-tdTomato mice to produce LepRb-tdTomato reporter mice [14]. To examine the colocation of LepRb and BDNF, LepRb-ires-Cre mice were crossed with *Bdnf*^*klox/klox*^ mice. The *Bdnf*^*flox/+;Lepr-ires-Cre*^ offspring were used to detect the presence of β-galactosidase encoded by *lacZ*. To generate conditional knockout mice lacking *Bdnf* in Lepr neurons, *Bdnf*^*flox/flox*^ mice were crossed with LepRb-ires-Cre mice. The *Bdnf*^*flox/+;Lepr-ires-Cre*^ offspring were back-crossed with *Bdnf*^*flox/flox*^ to produce *Bdnf*^*flox/flox;Lepr-ires-Cre*^ and *Bdnf*^*flox/flox*^ littermates for the experiments. *Lepr*^*flox/flox;Emx1-Cre*^ mice were generated as previously described [13]. Briefly, Emx1-Cre mice were crossed with *Lepr*^*flox/flox*^ mice. The *Lepr*^*flox/+;Emx1-Cre*^offspring were subsequently crossed with *Lepr*^*flox/flox*^ mice to generate *Lepr*^*flox/flox*;*Emx1-Cre*^ mice and *Lepr*^*flox/flox*^ littermate controls. The PCR primers used for genotyping were as follows: LepRb-ires-Cre: forward-5′-GCGGTCTGGCAGTAAAAACTATC-3′, reverse-5′-GTG AAACAGCATTGCTGTCACTT-3′; *Bdnf*^*flox/flox*^: forward-5′-TGTGATTGTGTTTCTGGTGAC-3′, reverse-5′-GCCTTCATGCAACC GAAGTATG-3′; *Bdnf*^*klox/klox*^: forward-5′-CTTGGGTGGAGAGGCTATTC-3′, reverse-5′-AGGTGAGATGACAGGAGATC-3′; *ob/ob*: primer1: 5-GCAGTCGGTATCCGCCAAGCAG-3′, primer2: -5′-GTGGTCTACAGGAGGGAGAGAAATG-3′; SNP1: 5′-TAGCCAATGACCTGGAGA ATCACT-3′; SNP2: 5′-CCAGCAGATGGAGGAGGTCACG-3′; and *db/db*: primer1: 5′-TTTTTATTTTGCTTGCTTATTTTGT-3′, primer2: 5′-GATTTGAACTCAGGACCTTTGG-3′; SNP1:5′-GATGTTTACATTTTGATGGACGT-3′; SNP2: 5′-TCAAACCATAGTTTAGGTTTGTATC-3′; and Emx1-Cre: forward-5′-CACTCATGGAAAATAGCGATC-3′, reverse-5′-ATCTCCGGTATTGAAACTCCAGCGC-3′.

### Drugs

Recombinant mouse leptin (R&D Systems, Minneapolis, MN, USA) was dissolved in sterile saline and administered intraperitoneally (i.p.) at a dose of 1 or 5 mg/kg body weight. For intra-dentate gyrus injections, AKT inhibitor (AKTi) VIII (1,3-dihydro-1-(1-((4-(6-Phenyl-1H-imidazo[4,5-g]quinoxalin-7-yl)phenyl)methyl)-4-piperidinyl)-2H-benzimidazol-2-one), an isozyme-selective AKTi specific for AKT1/2 (Calbiochem, La Jolla, CA, USA), was dissolved in dimethylsulfoxide (Sigma-Aldrich, Saint Louis, MO, USA) and injected at a dose of 3.0 μg/μl. C646, a specific inhibitor of histone acetyltransferase p300 (p300 HAT; Sigma-Aldrich) was dissolved in dimethylsulfoxide and injected at a dose of 3 μg/μl.

### Immunohistochemistry

*Lepr-ires-cre*^*tdTomato*^ and *Bdnf*^*klox/+;Lepr-ires-cre*^ mice were transcardially perfused with 4% paraformaldehyde. The brains were removed, postfixed overnight and then cryoprotected in 30% sucrose and cut into 20-μm coronal sections. From serial coronal sections of the entire dorsal-ventral axis of dentate gyrus (from −1.34 to −3.52 mm posterior to bregma), every sixth section was selected from each animal and processed for immunohistochemical staining. The sections were rinsed three times in phosphate-buffered saline (PBS), and incubated in blocking buffer (1% bovine serum albumin, 3% goat serum, 0.3% Triton X-100 in PBS) for 1 h. The sections were then incubated with mouse anti-NeuN antibody (#ab104224, 1:500; Abcam, Cambridge, UK) and rabbit anti-β-galactosidase antibody (#ab4761, 1:1000, Abcam, Cambridge, UK) overnight at 4 °C. After washing in PBS, sections were incubated for 4 h with fluorescent secondary antibodies: Alexa Fluor® 488 donkey antimouse IgG (#A-21202, 1:400, Invitrogen, Carlsbad, CA, USA) and Alexa Fluor® 555 donkey antirabbit IgG (#A-31572, 1:400, Invitrogen, Carlsbad, CA, USA). Finally, the sections were washed in PBS, mounted onto poly-lysine-coated glass slides, cover-slipped using fluorescence mounting medium and visualized via an Olympus FV1000 confocal microscope (Olympus, Shinjuku, Tokyo, Japan). Cell numbers were quantified on every sixth section throughout the dorsal-ventral axis of hippocampus using unbiased stereology. LepRb-tdTomato-, β-galactosidase-, and NeuN-positive cells within both sides of the hippocampus were counted. Fluorescent cells that intersected the exclusion boundaries of the unbiased sampling frame were excluded from counting. Cells that met the counting criteria were counted bilaterally throughout the dentate gyrus. The colocalization of LepRb-tdTomato or β-galactosidase with NeuN was confirmed with z-stack of the respective cell soma using line-based sequential scan (4-μm interval). The percentage of NeuN-positive neurons that were also double-labeled for LepRb-tdTomato and β-galactosidase within the granule cell layer was calculated.

### Western blot assay

Mice were killed by rapid decapitation. The hippocampus was dissected out on ice and immediately homogenized in the lysis buffer with 1% phenylmethylsulfonyl fluoride and 1 × PhosSTOP phosphatase inhibitor cocktail (Roche Applied Science, Penzberg, Germany). The denatured protein was separated on an SDS-polyacrylamide gel electrophoresis and transferred to a polyvinylidene fluoride membrane. The membrane was blocked in a solution of Tris-buffered saline with 1% dried milk and 0.1% Tween 20. Membranes were then incubated with the following primary antibodies diluted in the blocking solution: anti-AKT (#9272, 1:1000, Cell Signaling Technology, Danvers, MA, USA), anti-phospho-AKT (Thr308) (#2965, 1:1000, Cell Signaling), anti-BDNF (sc-546, 1:500, Santa Cruz Biotechnology), anti-ERK1/2 (#9102, 1:1000, Cell Signaling), anti-phospho-ERK1/2 (Thr202/Tyr204) (#4370, 1:1000, Cell Signaling), anti-TrkB (#4603, 1:1000, Cell Signaling), anti-pTrkB (Tyr516) (#4619, 1:1000, Cell Signaling), anti-β-actin (#4970, 1:1000, Cell Signaling), anti-STAT3 (#9139, 1:1000, Cell Signaling), anti-p-STAT3 (#9145, 1:1000, Cell Signaling). After washing, membranes were incubated with goat antirabbit IR Dye 680LT (#926-68021, 1:5000, Li-COR Biosciences, Lincoln, NE, USA) or goat antimouse IR Dye 800CW (#926-32210, 1:5000, Li-COR Biosciences) fluorescent secondary antibodies secondary antibodies and visualized using an Odyssey infrared imaging system (Li-COR Biosciences).

### Reverse transcription (RT) and quantitative PCR

The hippocampus was isolated from the mouse brain as described above and dentate gyrus tissue was dissected from 100-μm-thick coronal brain slices (bregma: −1.34 mm to −3.52 mm). Total RNA was extracted from brain tissue to generate cDNA using a previously described protocol [52]. First, total RNA was treated with 4 × gDNA wiper mix at 42 °C for 2 min to remove genomic DNA contamination, then reverse transcribed into cDNAs with 5 × HiScript II QRT SuperMix that was added to the reaction mixture and incubated at 25 °C for 10 min followed by 50 °C for 30 min and 85 °C for 5 min. The resulting cDNA was used for real-time PCR detection using the StepOnePlus real-time PCR system (Applied Biosystems, Waltham, MA, USA). The condition for PCR was 95 °C for 5 min, followed by 40 cycles of 95 °C for 10 s and 60 °C for 30 s. The primer sequences used to amplify each desired product were as follows: *Bdnf* exon IX (forward: GCGCCCATGAAAGAAGTAAA; reverse: TCGTCAGACCTCTCGAACCT), exon I (forward: 5′-CCTGCATCTGTTGGG GAGAC-3′; reverse: 5′-GCCTTGTCCGTGGACGTTTA-3′), exon II (forward: 5′-CTAGCCACCGGGGTGGTGTAA-3′; reverse: 5′-AGGATGGTCATCACTCTTCTC-3′), exon III (forward: 5′-CTTCATTGAGCCCAGGTCC-3′; reverse: 5′-CCGTGGACGTTTACTTCTTTC-3′), exon IV (forward: 5′-CAGAGCAGCTGCCTTGATGTT-3′; reverse: 5′-GCCTTGTCCGTGGACGTTTA-3′), exon VI (forward: 5′-CTGGGAGGCTTTGATGAGAC-3′; reverse: 5′-GCCTTCATGCAACCGAAGTA-3′), Cre (forward: 5′-GATTTCGACCAGGTTCGTTC-3′; reverse: 5′- GCTAACCAGCGTTTTCGTTC-3′), and β-tubulin (forward: 5′-AGCAACATGAATGACCTGGTG-3′; reverse: 5′-GCTTTCCCTAACCTGCTTGG-3′). The housekeeping gene β-tubulin was used as a reference gene for normalization of gene expression. The 2^−ΔΔCT^ method, i.e., delta-delta-ct analysis, was used as a relative quantification.

### Chromatin immunoprecipitation (ChIP) analysis

The hippocampus was dissected out from the brain on ice and incubated in 1% formaldehyde in PBS for 10 min followed by incubation with 125 mM glycine for 10 min at room temperature. Tissues were then washed three times in PBS and homogenized in SDS lysis buffer (167 mM NaCl, 0.01% SDS, 1.1% Triton X-100, 1.2 mM EDTA, 16.7 mM Tris-HCl, pH 8.1) containing PhosSTOP Phosphatase Inhibitor Cocktail (Roche Applied Science, Penzberg, Germany). Tissue samples were sonicated on ice using a sonicator (FB-705; Fisher Scientific) at 50% amplitude, 5 s on/10 s off, for 5 min. Samples were then centrifuged at 12,000 rpm for 10 min at 4 °C, and supernatants were collected for dilution in the ChIP dilution buffer (167 mM NaCl, 0.01% SDS, 1.1% Triton X-100, 1.2 mM EDTA, 16.7 mM Tris-HCl, pH 8.1). Then, the resulting chromatin solution was precleared with 60 µ salmon sperm DNA/protein A-agarose (EMD Millipore, Burlington, MA, USA) before binding with mouse IgG (#12-371, Sigma-Aldrich) or rabbit IgG (#12-370, Sigma-Aldrich) for 3–4 h at 4 °C and centrifuged for 2 min at 3000 rpm. Immunoprecipitations were performed at 4 °C overnight with primary antibodies: antiacetyl H3 (#06–599, EMD Millipore), antiacetyl H4 (#06-866, Millipore), anti-H3K4me2 (#ab7766, Abcam), anti-H3K9me2 (#ab1220, Abcam), anti-p300 HAT (#05–257, EMD Millipore), anti-H3K27me2 (#ab24684, Abcam). DNA-histone complex was collected by binding to salmon sperm DNA blocked-protein A-agarose beads for a 3–4 h incubation at 4 °C and sequentially washed with low salt buffer (150 mM NaCl, 0.1% SDS, 1% Triton X-100, 2 mM EDTA, 20 mM Tris-HCl, pH 8.1), high salt buffer (500 mM NaCl, 0.1% SDS, 1% Triton X-100, 2 mM EDTA, 20 mM Tris-HCl, pH 8.1), LiCl immune complex buffer (0.25 M LiCl, 1% IGEPAL-CA630, 1% deoxycholic acid, 1 mM EDTA, 10 mM Tris, pH 8.1) and Tris-EDTA buffer (10 mM Tris-HCl, 1 mM EDTA, pH 8.0). DNA-histone complexes were removed from protein A-agarose beads by incubation with elution buffer (0.1 M NaHCO_3_, 1% SDS) for 15 min at room temperature followed by incubation with NaCl (final concentration 0.2 M) overnight at 65 °C to dissociate DNA and histones. After proteinase K digestion for 2 h at 45 °C, DNA was extracted using DNA extraction Kits (Omega bio-tek, Doraville, GA, USA). Immunoprecipitated DNA was subjected to quantitative real-time PCR using primers specific to the mouse *Bdnf* promoters or GAPDH: *Bdnf* promoter I: forward-5′-TGATCATCACTCACGACCACG-3′, Reverse-5′- CAGCCTCTCTGAGCCAGTTACG-3′; *Bdnf* promoter II: forward-5′-CCGTCTTGTATTCCATCCTTTG-3′, Reverse-5′-CCCAACTCCACCACTATCCTC-3′; *Bdnf* promoter III: forward-5′-GTGAGAACCTGGGGCAAATC-3′, Reverse-5′-ACGGAAAAGAGGGAGGGAAA-3′; *Bdnf* promoter IV: forward-5′-CTTCTGTGTGCGTGAATTTGCT-3′, Reverse-5′-AGTCCACGAGAGGGCTCCA-3′; *Bdnf* promoter VI: forward-5′-ACTCACACTCGCTTCCTCCT-3′, Reverse-5′-GCACTGGCTTCTCTCCATTT-3′; *GAPDH*: forward-5′-CTCCCAGGAAGACCCTGCTT-3′, Reverse-5′-GGAACAGGGAGGAGCAGAGA-3′.The GAPDH promoter served as an internal gene control.

### DNA methylation analysis

DNA was extracted from hippocampal tissue and each sample (300 ng) was processed for bisulfite conversion using the EZ DNA Methylation Kit (Zymo Research, Orange, CA, USA). The bisulfite-converted samples were amplified using specific primers for the CpG islands that were selected from the promoter and exonic regions of the *Bdnf* gene for methylation analysis. The primers used to amplify six-specific CpG island regions were as follows: CpG1: forward-5′-GGGAAGTATTTAAAATAGGGTAG-3′, Reverse-5′-GGGAAGTATTTAAAATAGGGTAG-3′; CpG2: forward-5′-TTATTTAGTATTTTGGATAGAGTTAG-3′, Reverse-5′- CAATAAAAAAACCAAACTAAAACTC-3′, CpG3: forward-5′- TTTATAAAGTATGTAATGTTTTGGAA-3′, Reverse -5′- TACTCCTATTCTACAACAAAAAAATTAAAT-3′; CpG4: forward-5′- GGTATAGAGTTTTGGGTTTAAGTAG-3′, Reverse -5′- AAAATCAAACATTATTTAACTCTTC-3′; CpG5: forward-5′- TAGTGTTTGGTTTTGGTTGAGTTT-3′, Reverse -5′- CTACCCCAAAACAATAATAACAATTAAA-3′; and CpG6: forward-5′- ATTGTTATTATTGTTTTGGGGTAGA-3′, Reverse -5′- AAATTCTACAATCCCACAACTTCTC-3′. The thermocycler protocol involved an initial denaturation cycle (5 min, 95 °C), 40 cycles of denaturation (45 s, 95 °C), annealing (30 s, 56–58 °C), and extension (30 s, 72 °C), followed by a final extension cycle (5 min, 72 °C) terminating at 4 °C. The PCR products were then purified using a gel extraction kit (Qiagen, Germantown, MD, USA). The methylated CpG sites were detected using a PyroMark Q96 MD Pyrosequencing System (Qiagen) at the University of Texas Health Science Center at San Antonio Core Facility. The percentage of methylation of each CpG site within the region amplified was calculated by the MultiExperiment Viewer.

### Intra-dentate gyrus microinjection

For the deletion of the *Bdnf* gene selectively in the dentate gyrus, anesthetized adult *Bdnf*^*flox/flox*^ mice underwent bilateral stereotaxic injections of AAV-Cre-GFP or AAV-GFP (0.5 μl /side; Vector Biolabs, Malvern, PA) into the dentate gyrus (coordinates: AP = −2.1 mm, ML = ±1.5 mm, DV = −2.3 mm from Bregma), at a rate of 0.1 μL/min with a 33-gauge stainless steel injector connected to a UMP3 micro syringe pump (World Precision Instruments, Sarasota, FL). Additional 5 min were allowed for diffusion and prevention of backflow. Behavioral experiments were conducted 21 days after AAV injection. For intra-dentate gyrus microinjection of inhibitors of AKT and p300 HAT, the mice were anesthetized with 4% chloral hydrate (400 mg/kg, i.p.). AKTi and C646 (0.5 μl/side) were injected bilaterally into the dentate gyrus of adult C57BL/6J mice at a rate of 0.5 μl/min with a 33-gauge stainless steel injector. Additional 5 min were allowed for diffusion and prevention of backflow, and 30 min later, mice were injected with leptin (5 mg/kg, i.p.).

### Estrous cycle

The stages of the estrous cycle were monitored by analysis of cell types in vaginal lavages. Vaginal lavages were collected daily between 7:00 and 8:00 a.m. as described previously [53]. Briefly, vaginal fluid was placed on slides, and the slides were stained with crystal violet. The types of cells in vaginal smears were examined under a light microscope. Diestrus was defined by the presence of small leukocytes, and proestrus was defined by the presence of clumps of large, round, nucleated epithelial cells.

### Forced swim test

Mice were placed into a clear Plexiglas cylinder (25 cm in height and 10 cm in diameter) filled with water (24 °C) to a depth of 15 cm. A 6-min swim session was videotaped by a camera mounted above the cylinder. The duration of immobility was measured for the last 4 min. Immobility was defined as the absence of all movements except those required for respiration.

### Tail suspension test

The apparatus consisted of a box (30 × 30 × 30 cm) with an open front and a bar placed horizontally 1 cm from the top with an attached vertical bar hanging down in the center. Mice were individually suspended by the tail on the vertical bar with adhesive tape affixed 2 cm from the tip of the tail. A camera positioned in front of the box was used to record the animals’ behavior for a 6-min test session. Immobility in this test was defined as the absence of any limb or body movements, except those caused by respiration.

The behaviors of each mouse were scored by experimenters who were blinded to the genotypes or treatment conditions.

### Statistical analysis

All statistical analyses were performed using the statistical software GraphPad Prism 8. Shapiro–Wilk test and *F*-test were used to test normality and equal variance assumptions, respectively. For normally distributed data, two-tailed *t*-tests were used to assess differences between two experimental groups with equal variance. For normally distributed data with unequal variances, two-tailed *t*-tests with Welch’s correction were used. One-way analyses of variance (ANOVAs) followed by Sidak post hoc tests were used for analysis of three or more groups. For nonnormally distributed data, Mann–Whitney *U* tests were performed to compare two groups. For analysis of three or more groups with nonnormally distributed data, the Kruskal–Wallis test followed by Dunn’s multiple comparisons test was used. Two-way or three-way ANOVAs followed by Bonferroni tests were used where appropriate. *P* < 0.05 was considered significant.

## Results

### Colocalization of LepRb and BDNF in the dentate gyrus of the hippocampus

**Fig. 1:**
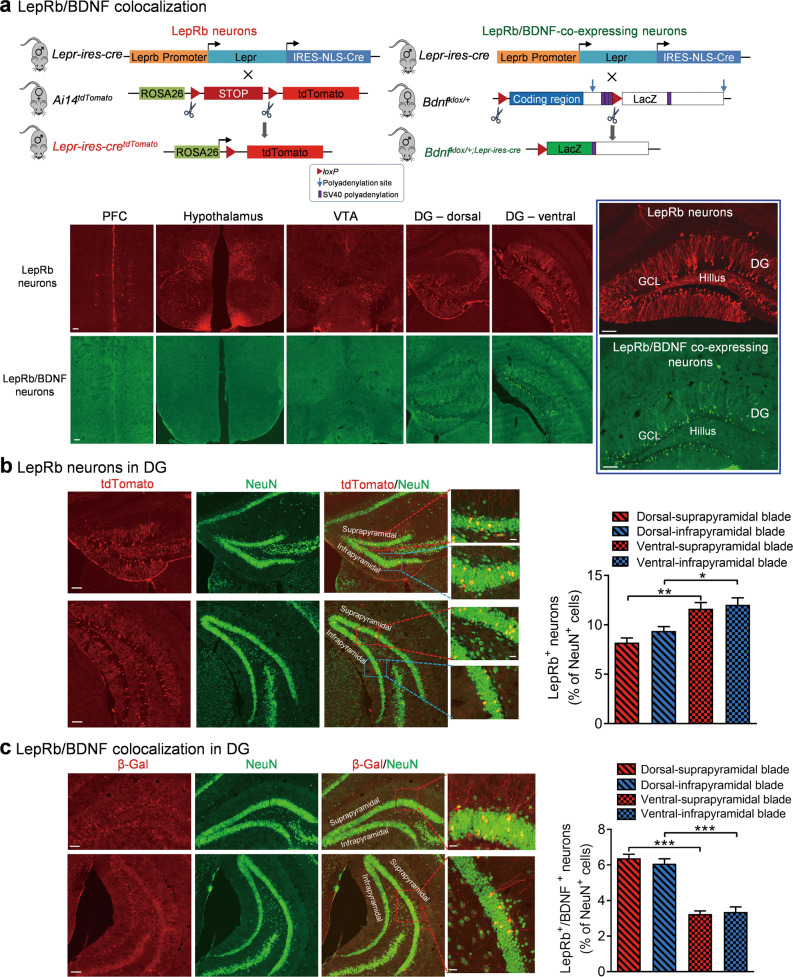
LepRb/BDNF colocalization in the dentate gyrus of the hippocampus. **a** Colocalization of LepRb and BDNF. Upper panel: schematic diagram of the strategy used for generation of tdTomato^Lepr-ires-Cre^ (left) and *Bdnf*^*klox/+;LepR-ires-Cre*^ mice (right). Lower panel: representative confocal images showing LepRb-expressing neurons labeled with tdTomato (red) in LepRb-Cret^dTomato^ mice and neurons coexpressing LepRb and BDNF labeled with β-galactosidase (green) in *Bdnf*^*klox/+;LepR-ires-Cre*^ mice. Sale bars = 100 μm. DG dentate gyrus; GCL granule cell layer; PFC prefrontal cortex; VTA ventral tegmental area. **b** Distribution of LepRb-expressing neurons in the dentate gyrus. Left, representative images showing LepRb-expressing neurons (red) and NeuN-positive cells (green) in the dentate gyrus. Right, quantitative analysis of dentate gyrus neurons expressing LepRb in the suprapyramidal versus infrapyramidal blade and in the dorsal versus ventral region. *n* = 4 mice. **c** Distribution of neurons coexpressing LepRb and BDNF in the dentate gyrus. Left, representative images showing β-galactosidase-positive cells, indicative of LepRb/BDNF-coexpressing neurons (red), and NeuN-positive cells (green). Right, quantitative analysis of dentate gyrus neurons coexpressing LepRb and BDNF in the subcompartments of the dentate gyrus. *n* = 4 mice. Sale bars = 100 μm for low magnification images and 20 μm for high magnification images. *P < 0.05; **P < 0.01; ***P < 0.001.

While alternative splicing produces multiple isoforms of the LepR, the long isoform, LepRb, has an intracellular domain that is essential for signal transduction required to elicit the physiological effects of leptin [54, 55]. We examined the distribution of LepRb-expressing neurons in the brain using LepRb-ires-Cre × Ai14-tdTomato (LepRb-tdTomato) reporter mice, in which tdTomato marks LepRb-expressing cells [4, 14]. As shown previously [4, 14, 56], leptin target neurons were found in the hypothalamus, prefrontal cortex, hippocampus, and ventral tegmental area (Fig. 1a). To examine if these neurons express BDNF, we crossed LepR-ires-Cre mice with *Bdnf*^*klox/+*^ mice, in which BDNF neurons express β-galactosidase once the floxed *Bdnf* allele is deleted by Cre-mediated recombination [51, 57]. In *Bdnf*^*klox/+;LepR-ires-Cre*^ mice, very few β-galactosidase-expressing neurons were detected in the hypothalamus, prefrontal cortex, or ventral tegmental area, indicating lack of co-expression between LepRb and BDNF in these brain regions. However, dense populations of granule neurons in the dentate gyrus of the hippocampus expressed β-galactosidase (Fig. 1a), suggesting the co-expression of LepRb and BDNF.

The dentate gyrus is composed of the suprapyramidal and infrapyramidal blades, and the dorsal and ventral regions are functionally distinct [58]. Therefore, we analyzed the distribution of neurons expressing LepRb alone and coexpressing LepRb/BDNF in these subcompartments of the dentate gyrus. In LepRb-tdTomato reporter mice, LepRb neurons were labeled with tdTomato. We quantified the numbers of LepRb neurons and total NeuN-positive cells in the suprapyramidal versus infrapyramidal blade and in the dorsal versus ventral region. Data analysis revealed that a higher proportion of neurons in the ventral region expressed LepRb (blade: *F*_(1,12)_ = 1.8970, *P* = 0.1936; region: *F*_(1,12)_ = 28.4700, *P* < 0.001; total number of LepRb neurons counted, 2380; total number of NeuN-positive neurons counted, 27,155 from 4 mice) (Fig. 1b). Furthermore, we quantified the numbers of β-galactosidase-positive cells, indicative of colocalization of LepRb and BDNF, and total NeuN-positive cells in *Bdnf*^*klox/+;LepR-ires-Cre*^ mice. Cell counting results indicated a region-specific distribution with a higher percentage of neurons in the dorsal region expressing both LepRb and BDNF (blade: *F*_(1,12)_ = 0.1587, *P* = 0.6973; region: *F*_(1,12)_ = 144.5000, *P* < 0.001; total number of LepRb/BDNF neurons counted, 2029; total number of NeuN-positive neurons counted, 37,318 from 4 mice) (Fig. 1c). By comparing the numbers and the distribution of LepRb-expressing neurons with LepRb/BDNF-colocalized neurons in the dentate gyrus, we determined that a high proportion of LepRb-expressing neurons (up to 79%) contain detectable BDNF especially in the dorsal region, which provide an anatomical basis to study functional interactions between LepRb signaling and BDNF.

### Leptin regulates BDNF mRNA and protein expression in the hippocampus

**Fig. 2:**
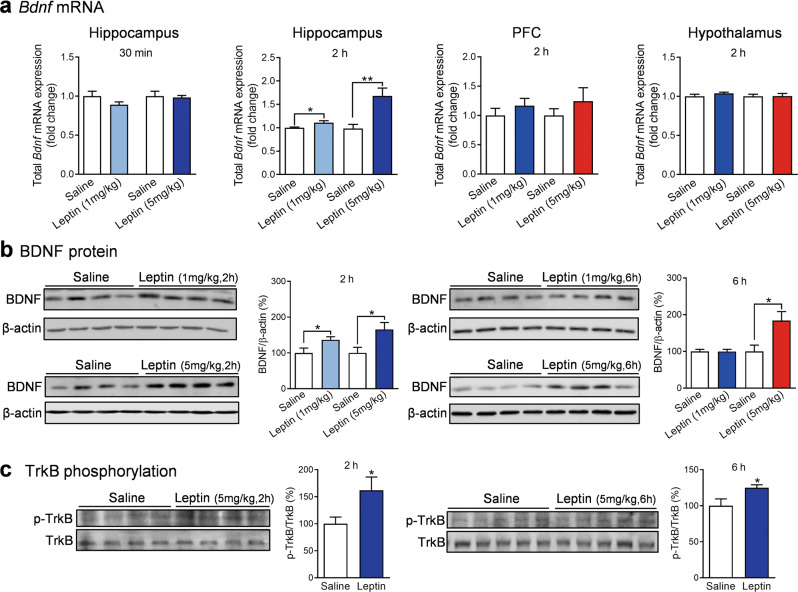
Regulation of BDNF expression and TrkB activation by leptin. **a** Total (exon IX) *Bdnf* mRNA levels in the hippocampus, prefrontal cortex (PFC), and hypothalamus at 30 min or 2 h after leptin injection (1 mg/kg or 5 mg/kg, i.p.). Hippocampus-30 min: saline, *n* = 7; leptin (1 mg/kg), *n* = 6; saline, *n* = 7; leptin (5 mg/kg), *n* = 6. Hippocampus-2 h: saline, *n* = 12; leptin (1 mg/kg), *n* = 11; saline, *n* = 10; leptin (5 mg/kg), *n* = 11. PFC-2 h: saline, *n* = 6; leptin (1 mg/kg), *n* = 5; saline, *n* = 11; leptin (5 mg/kg), *n* = 12. Hypothalamus-2 h: saline, *n* = 6; leptin (1 mg/kg), *n* = 5; saline, *n* = 6, leptin (5 mg/kg), *n* = 7. **b** BDNF protein levels in hippocampus in response to leptin treatment. Post-injection 2 h: saline, *n* = 6; leptin (1 mg/kg), *n* = 6; saline, *n* = 6, leptin (5 mg/kg), *n* = 7. Post-injection 6 h: saline, *n* = 6; leptin (1 mg/kg), *n* = 6; saline, *n* = 5; leptin (5 mg/kg), *n* = 5. **c** Phosphorylated TrkB levels in the hippocampus at 2 and 6 h after leptin injection (5 mg/kg). Post-injection 2 h: saline, *n* = 6; leptin, *n* = 5. Post-injection 6 h: saline, *n* = 5; leptin, *n* = 5. *P < 0.05; **P < 0.01 compared with saline-injected controls.

We next examined whether leptin regulates *Bdnf* mRNA and protein expression. Specifically, we measured *Bdnf* exon IX mRNA levels using quantitative real-time PCR. The detection of exon IX allows for the evaluation of all isoforms transcribed, i.e., total *Bdnf* transcription. Wild-type mice received a single i.p. injection of leptin (1 or 5 mg/kg), and total *Bdnf* mRNA levels in the brain were measured 30 min or 2 h post injection. Leptin treatment caused no significant change in total *Bdnf* mRNA expression in the hippocampus at 30 min post injection (1 mg/kg, *t*_(11)_ = 1.4330, *P* = 0.1797; 5 mg/kg, *t*-test with Welch’s correction, *P* = 0.8218) (Fig. 2a); however, an increase was observed at 2 h post injection (1 mg/kg, *t*-test with Welch’s correction, *P* = 0.0272; 5 mg/kg, Mann–Whitney test, *P* = 0.0011) (Fig. 2a). In contrast, total *Bdnf* mRNA levels in the PFC and hypothalamus remained unaltered 2 h following the same leptin treatments (PFC, 1 mg/kg, Mann–Whitney test, *P* = 0.1255; 5 mg/kg, Mann–Whitney test, *P* = 0.8328; hypothalamus, 1 mg/kg, Mann–Whitney test, *P* = 0.1775; 5 mg/kg, Mann–Whitney test, *P* = 0.6282) (Fig. 2a). In parallel, BDNF protein levels in the hippocampus were increased 2 h after leptin treatment (1 or 5 mg/kg) (1 mg/kg, *t*_(10)_ = 2.2810, *P* = 0.0457; 5 mg/kg, *t*_(11)_ = 2.5670, *P* = 0.0262) (Fig. 2b). The increase in BDNF protein levels persisted at least 6 h after an i.p. injection of 5 mg/kg of leptin (*t*_(8)_ = 2.8310, *P* = 0.0221). However, this was not observed after an i.p. injection of a lower dose of leptin (1 mg/kg) (*t*_(10)_ = 0.0470, *P* = 0.9634) (Fig. 2b).

Most biological actions of BDNF are transduced through TrkB receptors [27, 59, 60]. Therefore, we next examined whether increased BDNF production would lead to activation of TrkB, which was assessed by its tyrosine phosphorylation [61]. A single leptin injection (5 mg/kg, i.p.) increased phosphorylated TrkB levels in the hippocampus at 2 and 6 h post injection (2 h, *t*_(9)_ = 2.3740, *P* = 0.0417; 6 h, *t*_(8)_ = 2.4050, *P* = 0.0428) (Fig. 2c), suggesting that leptin induces activation of the BDNF-TrkB signaling pathway.

### Regulation of exon-specific *Bdnf* mRNA expression by leptin treatment, leptin deficiency and leptin receptor deficiency

**Fig. 3:**
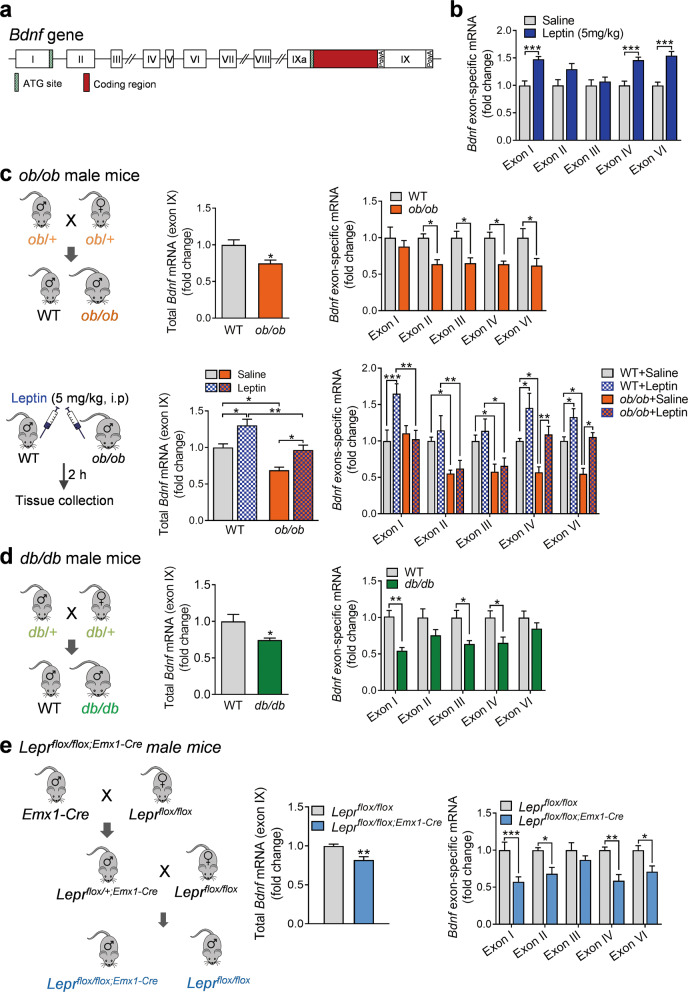
Regulation of exon-specific *Bdnf* mRNA expression by leptin signaling. **a** The structure of the mouse *Bdnf* gene. **b** Wild-type mice. Exon-specific *Bdnf* mRNA expression levels. Saline, *n* = 16; leptin (5 mg/kg, i.p.), *n* = 17. **c** *ob/ob* mice. Upper panel: left, schematic diagram of breeding strategy; middle, total *Bdnf* mRNA; right, *Bdnf* exon-specific mRNA. WT, *n* = 6, *ob/ob*, *n* = 6. Lower panel: left, schematic diagram of breeding strategy and leptin treatment; middle, total *Bdnf* mRNA; right, Bdnf exon-specific mRNA. *n* = 5 per group. **d** *db/db* mice. Left, schematic diagram of breeding strategy; middle, total *Bdnf* mRNA; right, *Bdnf* exon-specific mRNA. WT, *n* = 5; *db/db*, *n* = 5. **e** Lepr conditional knockout mice. Left, schematic diagram of breeding strategy; middle, total *Bdnf* mRNA; right, *Bdnf* exon-specific mRNA. *Lepr*^*flox/flox*^, *n* = 7; *Lepr*^*flox/flox;Emx1-Cre*^, *n* = 7. *P < 0.05; **P < 0.01; ***P < 0.001 compared with wild-type littermates or saline-injected controls.

Leptin-induced changes in total (exon IX) *Bdnf* mRNA levels could be related to the changes in noncoding exons I–VIII spliced to exon IX (Fig. 3a). To determine which noncoding exons may directly contribute to the modulation of total *Bdnf* expression by leptin, we analyzed exons I, II, III, IV, and VI that are highly expressed in the hippocampus. Exons V, VII, and VIII were not included due to their very low expression levels (data not shown). While mRNA levels of *Bdnf* exons II and III remained unchanged, exons I, IV, and VI transcripts were found to increase 2 h after leptin treatment (5 mg/kg) (treatment: *F*_(1,155)_ = 52.8300, *P* < 0.001; exon: *F*_(4,155)_ = 2.8020, *P* = 0.0278; treatment × exon interaction: *F*_(4,155)_ = 2.8020, *P* = 0.0278; exons I: *P* < 0.001; exons II: *P* = 0.0511; exons III: *P* > 0.9999; exons IV: *P* < 0.001; exons VI: *P* < 0.001) (Fig. 3b). These results suggest that *Bdnf* transcripts containing exons I, IV, and VI are responsible for leptin-induced upregulation of total *Bdnf* gene expression in the hippocampus.

We then asked whether deficiency of leptin or the leptin receptor represses *Bdnf* mRNA expression. We investigated the modulation of total *Bdnf* mRNA (exon IX) in the hippocampus as compared to mRNA levels of noncoding exons. Leptin-deficient (*ob/ob*) mice showed decreased total *Bdnf* mRNA expression (*t*_(10)_ = 3.0790, *P* = 0.0117) (Fig. 3c). Analysis of exon-specific *Bdnf* expression revealed that mRNAs for exons II, III, IV, and VI were downregulated in *ob/ob* mice (genotype: *F*_(1,50)_ = 31.0300, *P* < 0.001; exon: *F*_(4,50)_ = 0.7510, *P* = 0.5620; genotype × exon interaction: *F*_(4,50)_ = 0.7510, *P* = 0.5620, exons I: *P* > 0.9999; exons II: *P* = 0.0305; exons III: *P* = 0.0412; exons IV: *P* = 0.0303; exons VI: *P* = 0.0197) (Fig. 3c). We next determined whether leptin replenishment in *ob/ob* mice can restore abnormal *Bdnf* expression. *Ob/ob* mice and WT littermates received a single i.p. injection of leptin (5 mg/kg) 2 h prior to dissection of hippocampal tissue. Leptin replacement rescued the downregulated expression of total *Bdnf* mRNA in ob/ob mice (*P* = 0.0381), comparable to the level seen in WT littermates (*P* = 0.0205) (genotype: *F*_(1,16)_ = 27.0900, *P* < 0.001; treatment: *F*_(1,16)_ = 21.5900, *P* < 0.001; genotype × treatment interaction: *F*_(1,16)_ = 0.0433, *P* = 0.8377) (Fig. 3c). mRNA expression of exons IV and VI, but not exons I, II, and III, was restored by leptin (treatment: *F*_(1, 80)_ = 29.7200, *P* < 0.001; genotype: *F*_(1,80)_ = 57.6200, *P* < 0.001; exon: *F*_(4,80)_ = 6.7280, *P* < 0.001; genotype × treatment × exon interaction: *F*_(4,80)_ = 2.3570, *P* = 0.0605) (Fig. 3c). The failure of leptin to reverse the downregulation of exons I, II, and III suggests that their repression is not a direct result of leptin deficiency.

The above results indicate that leptin deficiency decreases total *Bdnf* mRNA expression via downregulation of specific *Bdnf* exons. To confirm that lack of LepRb would exert similar effects, we analyzed *Bdnf* gene expression in *db/db* mice that lack intracellular leptin action through LepRb [55, 62]. As seen in *ob/ob* mice, total *Bdnf* mRNA (exon IX) expression was decreased in *db/db* mice (*t*-test with Welch’s correction, *P* = 0.0477) (Fig. 3d). Among other exons detected, mRNAs for exons I, III, and IV were downregulated (Fig. 3d) (genotype: *F*_(1,40)_ = 35.7800, *P* < 0.001; exon: *F*_(4,40)_ = 0.9118, *P* = 0.4664; treatment × exon interaction: *F*_(4,40)_ = 1.0480, *P* = 0.3949, exons I: *P* = 0.0015; exon II: *P* = 0.2189; exon III: *P* = 0.0183; exon IV: *P* = 0.0268; exon VI: *P* > 0.9999), which is distinct from that observed in *ob/ob* mice.

A global loss of leptin signaling in *ob/ob* and *db/db* mice causes obesity and type 2 diabetes [1, 8], which could contribute to the downregulation of *Bdnf* mRNA expression in the hippocampus. We have previously shown that forebrain-specific LepRb knockout (*Lepr*^*flox/flox;Emx1-Cre*^) mice have normal body weight and insulin levels [13], which have decreased LepRb mRNA expression in the hippocampus compared to WT (Lepr^flox/flox^: 1.00 ± 0.0778; Lepr^flox/flox;Emx1-Cre^: 0.46 ± 0.0695; *t*_(12)_ = 5.2030, *P* < 0.001). Therefore, we used *Lepr*^*flox/flox;Emx1-Cre*^ mice to examine the effect of loss of LepRb on *Bdnf* gene expression. In contrast to *ob/ob* and *db/db* mice showing increased body weight, we confirmed that *Lepr*^*flox/flox;Emx1-Cre*^ mice had normal body weight (*Lepr*^*flox/flox*^: 26.86 ± 0.349 g; *Lepr*^*flox/flox;Emx1-Cre*^: 26.93 ± 0.308 g; *t*_(12)_ = 0.1534, *P* = 0.8807). Total *Bdnf* mRNA expression was reduced in *Lepr*^*flox/flox;Emx1-Cre*^ mice (*t*_(12)_ = 3.7760, *P* = 0.0026) (Fig. 3e), accompanied by distinct exon-specific expression patterns with downregulation of *Bdnf* exon I, II, IV, and VI expression (genotype: *F*_(1,60)_ = 44.6700, *P* < 0.001; exon: *F*_(4,60)_ = 1.2560, *P* = 0.2971; treatment × exon interaction: *F*_(4,60)_ = 1.2560, *P* = 0.2971, exon I: *P* < 0.001; exon II: *P* = 0.0192; exon III: *P* > 0.9999; exon IV: *P* = 0.0012; exon VI: *P* = 0.0391) (Fig. 3e). These results indicate that the three lines of mutant mice lacking leptin signaling displayed a similar reduction of total *Bdnf* mRNA expression but different exon-specific downregulation patterns.

**Fig. 4:**
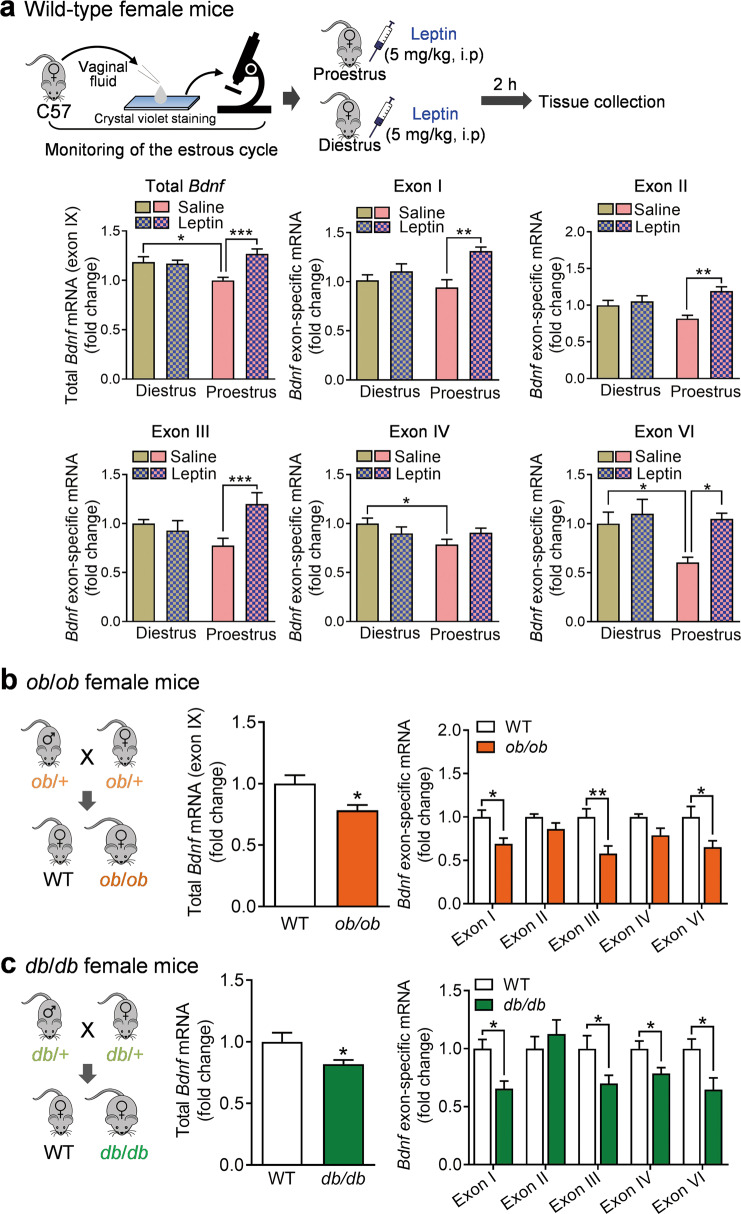
Regulation of exon-specific *Bdnf* mRNA expression by leptin in female mice. **a** Wild-type female mice. Upper panel, schematic diagram depicting the identification of the estrous cycle and leptin treatment. Middle and lower panels, total *Bdnf* and exon-specific mRNA expression levels in proestrus versus diestrus in response to leptin injection (5 mg/kg, i.p.). *n* = 11 per group. **b** *ob/ob* female mice. Left, schematic diagram of breeding strategy; middle, total *Bdnf* mRNA; right, *Bdnf* exon-specific mRNA. *n* = 6 per group. **c** *db/db* female mice. Left, schematic diagram of breeding strategy; middle, total *Bdnf* mRNA; right, *Bdnf* exon-specific mRNA. *n* = 6  per group. *P < 0.05; **P < 0.01; ***P < 0.001 compared with WT or saline-injected controls.

All experiments described above were conducted in male mice. We also examined the effects of leptin and deficiency in leptin or LepRb on *Bdnf* gene expression in female mice. As we have previously demonstrated that leptin induces AKT phosphorylation and antidepressant-like effects in the proestrus phase but not in the diestrus phase [14], we therefore tested the effects of leptin on *Bdnf* gene expression during these two stages of the estrous cycle. The estrous cycle was identified based on vaginal lavage prior to the treatments. Female mice in proestrus or diestrus received an i.p. injection with either vehicle (saline) or leptin (5.0 mg/kg), and 2 h later hippocampal tissue was collected for the measurement of mRNA levels. Leptin increased total *Bdnf* mRNA expression in the proestrus phase but not in the diestrus phase (treatment: *F*_(1,40)_ = 8.3640, *P* = 0.0062; estrus cycle: *F*_(1,40)_ = 0.9669, *P* = 0.3314; treatment × estrus cycle interaction: *F*_(1,40)_ = 10.3400, *P* = 0.0026) (Fig. 4a). Correspondingly, mRNA levels for exons I, II, III, and VI were increased in proestrus female mice but not in diestrus female mice (exon I: treatment: *F*_(1,40)_ = 12.4800, *P* = 0.0011; estrus cycle: *F*_*(*1,40)_ = 1.0630, *P* = 0.3087; treatment × estrus cycle interaction: *F*_(1,40)_ = 4.5870, *P* = 0.0384; exon II: treatment: *F*_(1,40)_ = 11.7200, *P* = 0.0014; estrus cycle: *F*_(1,40)_ = 0.1099, *P* = 0.7420; treatment × estrus cycle interaction: *F*_(1,40)_ = 6.6790, *P* = 0.0135; exon III: treatment: *F*_(1,40)_ = 4.0010, *P* = 0.0523; estrus cycle: *F*_(1,40)_ = 0.0719, *P* = 0.7899; treatment × estrus cycle interaction: *F*_(1,40)_ = 7.9640, *P* = 0.0074; exon IV: treatment: *F*_(1,40)_ = 0.0296, *P* = 0.8642; estrus cycle: *F*_(1,40)_ = 3.4420, *P* = 0.0709; treatment × estrus cycle interaction: *F*_*(*1,40)_ = 3.9090, *P* = 0.0550; exon IV: treatment: *F*_(1,40)_ = 0.0296, *P* = 0.8642; estrus cycle: *F*_(1,40)_ = 3.4420, *P* = 0.0709; treatment × estrus cycle interaction: *F*_(1,40)_ = 3.9090, *P* = 0.0550; exon VI: treatment: *F*_(1,40)_ = 7.0160, *P* = 0.0115; estrus cycle: *F*_(1,40)_ = 4.7560, *P* = 0.0351; treatment × estrus cycle interaction: *F*_(1,40)_ = 2.7830, *P* = 0.1031) (Fig. 4a). Furthermore, mRNA levels for total *Bdnf* and exons IV and VI varied across the estrous cycle with higher levels in the proestrus phase (total *Bdnf*, *P* = 0.0301; exons I: *P* = 0.8960; exons II: *P* = 0.9686; exons III: *P* = 0.2856; exon IV, *P* = 0.0467; exon VI, *P* = 0.0454) (Fig. 4a). These results suggest that basal expression levels of total *Bdnf* and specific exons and their responses to leptin treatment are estrous cycle-dependent, which could be partially due to a dynamic change in endogenous leptin and LepRb across the estrous cycle [63].

Both *ob/ob* and *db/db* female mice have impaired ovarian development [64]. They are infertile and exhibit no evidence of estrous cycles [65]. As seen in male mice, total *Bdnf* mRNA expression in the hippocampus was decreased in female *ob/ob* and *db/db* mice (*ob/ob*: *t*_(10)_ = 2.6750, *P* = 0.0233; *db/db*: *t*_(10)_ = 2.2510, *P* = 0.0481) with overlapping but distinct profiles of specific exon mRNA expression (Fig. 4b, c). While mRNAs for exons I, III, and VI were decreased in *ob/ob* female mice (genotype: *F*_(1,50)_ = 33.3600, *P* < 0.001; exon: *F*_(4,50)_ = 1.0160, *P* = 0.4083; genotype × exon interaction: *F*_(4,50)_ = 1.0160, *P* = 0.4083) (Fig. 4b), *db/db* female mice showed decreased levels of exons I, III, IV, and VI mRNAs (Fig. 4b, c) (genotype: *F*_(1,50)_ = 15.1900, *P* < 0.001; exon: *F*_(4,50)_ = 2.5860, *P* = 0.0480; genotype × exon interaction: *F*_(4,50)_ = 2.5860, *P* = 0.0480). These data suggest that the effects of leptin deficiency and leptin receptor deficiency on the transcription of *Bdnf* gene expression are sex-dependent.

### Leptin regulates *Bdnf* gene expression through epigenetic mechanisms

**Fig. 5:**
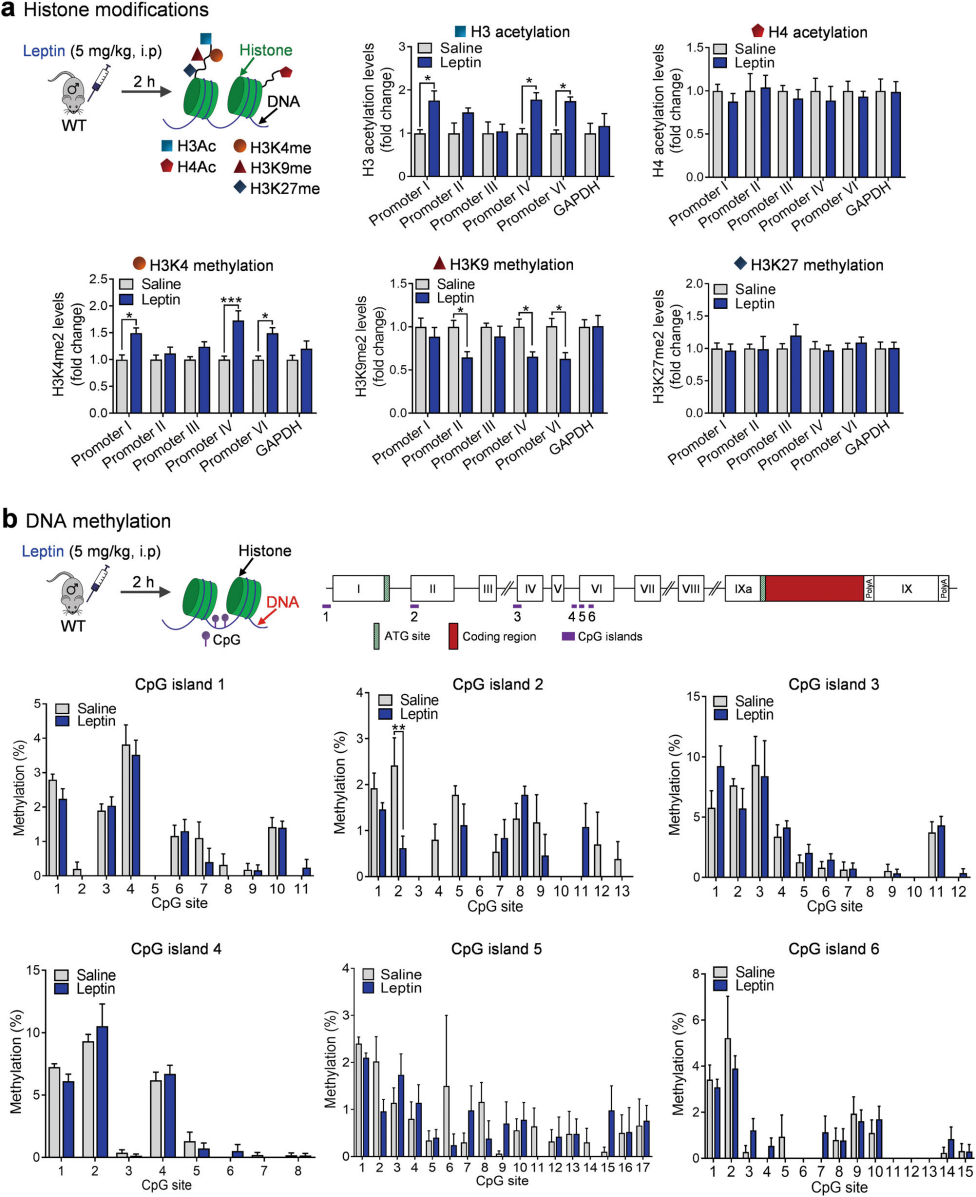
Epigenetic modifications of the *Bdnf* gene induced by leptin. **a** Upper left, schematic diagram of histone modifications. Upper middle, Histone H3 acetylation (*n* = 5 per group); upper right, histone H4 acetylation (*n* = 5 per group); lower left, H3K4 methylation (*n* = 8 per group); lower middle, H3K9 methylation (*n* = 8 per group); H3K27 methylation (*n* = 8 per group). **b** Upper panel, schematic diagram of the positions of Bdnf CpG islands 1–6. Middle and lower panels, percentage of methylation of each CpG site. DNA methylation levels of CpG1–CpG6, *n* = 5 per group. *P < 0.05; **P < 0.01; ***P < 0.001 compared with saline-injected controls.

We hypothesized that epigenetic modifications of the promoter region of *Bdnf* exons underlie leptin-induced regulation of *Bdnf* gene expression. To test this hypothesis, we first examined whether histone modifications at specific *Bdnf* promoters are altered in the hippocampus following leptin treatment. ChIP assays were performed at 2 h after leptin injection (5 mg/kg, i.p.) to quantify the relative levels of acetylated H3 and H4 at the promoter of exons I, II, III, IV, and VI (Fig. 5a). Histone H3 acetylation was greatly increased at promoters I, IV, and VI (treatment: *F*_(1,48)_ = 21.8300, *P* < 0.001; promoter: *F*_(5,48)_ = 1.5430, *P* = 0.3558; treatment × promoter interaction: *F*_(5,48)_ = 1.5430, *P* = 0.1942, promoter I: *P* = 0.0325; promoter II: *P* = 0.4199; promoter III: *P* > 0.9999; promoter IV: *P* = 0.0256; promoter VI: *P* = 0.0377; GAPDH: *P* > 0.9999), while no change in histone H4 acetylation was detected at any promoter (treatment: *F*_(1,48)_ = 0.7230, *P* = 0.3994; promoter: *F*_(5,48)_ = 0.1289, *P* = 0.9851; treatment × promoter interaction: *F*_(5,48)_ = 0.1289, *P* = 0.9851, promoter I: *P* > 0.9999; promoter II: *P* > 0.9999; promoter III: *P* > 0.9999; promoter IV: *P* > 0.9999; promoter VI: *P* > 0.9999; GAPDH: *P* > 0.9999) (Fig. 5a). In addition, we examined methylation of lysine 4, 9, and 27 on histone H3 at the *Bdnf* promoters. Leptin increased dimethylation of H3K4 at promoters I, IV, and VI (treatment: *F*_(1,84)_ = 41.0100, *P* < 0.001; promoter: *F*_(5,84)_ = 2.5460, *P* = 0.0340; treatment × promoter interaction: *F*_(5,84)_ = 2.5460, *P* = 0.0340, promoter I: *P* = 0.0062; promoter II: *P* > 0.9999; promoter III: *P* = 0.6081; promoter IV: *P* < 0.001; promoter VI: *P* = 0.0063; GAPDH: *P* = 0.9830), decreased dimethylation of H3K9 at promoters II, IV, and VI (treatment: *F*_(1,84)_ = 17.9800 *P* < 0.001; promoter: *F*_(5,84)_ = 1.6440, *P* = 0.1573; treatment × promoter interaction: *F*_(5,84)_ = 1.7060, *P* = 0.1422, promoter I: *P* > 0.9999; promoter II: *P* = 0.0348; promoter III: *P* > 0.9999; promoter IV: *P* = 0.0406; promoter VI: *P* = 0.0199; GAPDH: *P* > 0.9999), and had no effect on demethylation of H3K27 at any promoter (treatment: *F*_(1,84)_ = 0.3343, *P* = 0.5647; promoter: *F*_(5,84)_ = 0.3451, *P* = 0.8841; treatment × promoter interaction: *F*_(5,84)_ = 0.3451, *P* = 0.8841, promoter I: *P* > 0.9999; promoter II: *P* > 0.9999; promoter III: *P* > 0.9999; promoter IV: *P* > 0.9999; promoter VI: *P* > 0.9999; GAPDH: *P* > 0.9999) (Fig. 5a).

Alternatively, DNA methylation changes in the *Bdnf* gene region might mediate leptin-induced regulation of *Bdnf* gene expression in the hippocampus [66, 67]. Thus, we chose the CpG island of the *Bdnf* gene upstream of exon I (CpG1) and CpG islands within the promoters II, IV, and VI and the exonic regions of the *Bdnf* gene after the transcriptional start site of exons II, IV, and VI (CpG2–6) as targets for methylation analysis. Using bisulfite pyrosequencing, the methylation levels of CpG sites at *Bdnf* promoters/exon regions were quantified. No significant changes in methylated DNA levels associated with exons I, IV, and IV were observed after leptin treatment compared with vehicle controls (Fig. 5b), except for one out of 13 CpG sites within the promoter II CpG island, i.e., CpG island 2 (CpG1: treatment: *F*_(1,88)_ = 1.5180, *P* = 0.2211; CpGs: *F*_(10,88)_ = 37.9300, *P* < 0.001; treatment × CpG interaction: *F*_(10,88)_ = 0.5878, *P* = 0.8199; CpG2: treatment: *F*_(1,104)_ = 4.6230, *P* = 0.0339; CpGs: *F*_(12,104)_ = 7.4440, *P* < 0.001; treatment × CpG interaction: *F*_(12,104)_ = 2.3690, *P* = 0.0097; CpG3: treatment: *F*_(1,96)_ = 0.4993, *P* = 0.4815; CpGs: *F*_(11,96)_ = 18.1600, *P* < 0.001; treatment × CpG interaction: *F*_(11,96)_ = 0.7055, *P* = 0.7305; CpG4: treatment: *F*_(1,64)_ = 0.0011, *P* = 0.9740; CpGs: *F*_(7,64)_ = 83.0200, *P* < 0.001; treatment × CpG interaction: *F*_(7,64)_ = 0.7014, *P* = 0.6707; CpG5: treatment: *F*_(1,136)_ = 0.0775, *P* = 0.7812; CpGs: *F*_(16,136)_ = 3.0240, *P* < 0.001; treatment × CpG interaction: *F*_(16,136)_ = 1.0210, *P* = 0.4392; CpG6: treatment: *F*_(1,120)_ = 0.0880, *P* = 0.7673; CpGs: *F*_(14,120)_ = 12.3800, *P* < 0.001; treatment × CpG interaction: *F*_(14,120)_ = 0.7632, *P* = 0.7065). Our data indicate that the methylation of 75 out of 76 CpG sites that we analyzed was not sensitive to leptin treatment.

### Leptin-induced regulation of *Bdnf* mRNA expression is mediated by stimulation of an AKT/p300 HAT/H3 modification cascade

**Fig. 6:**
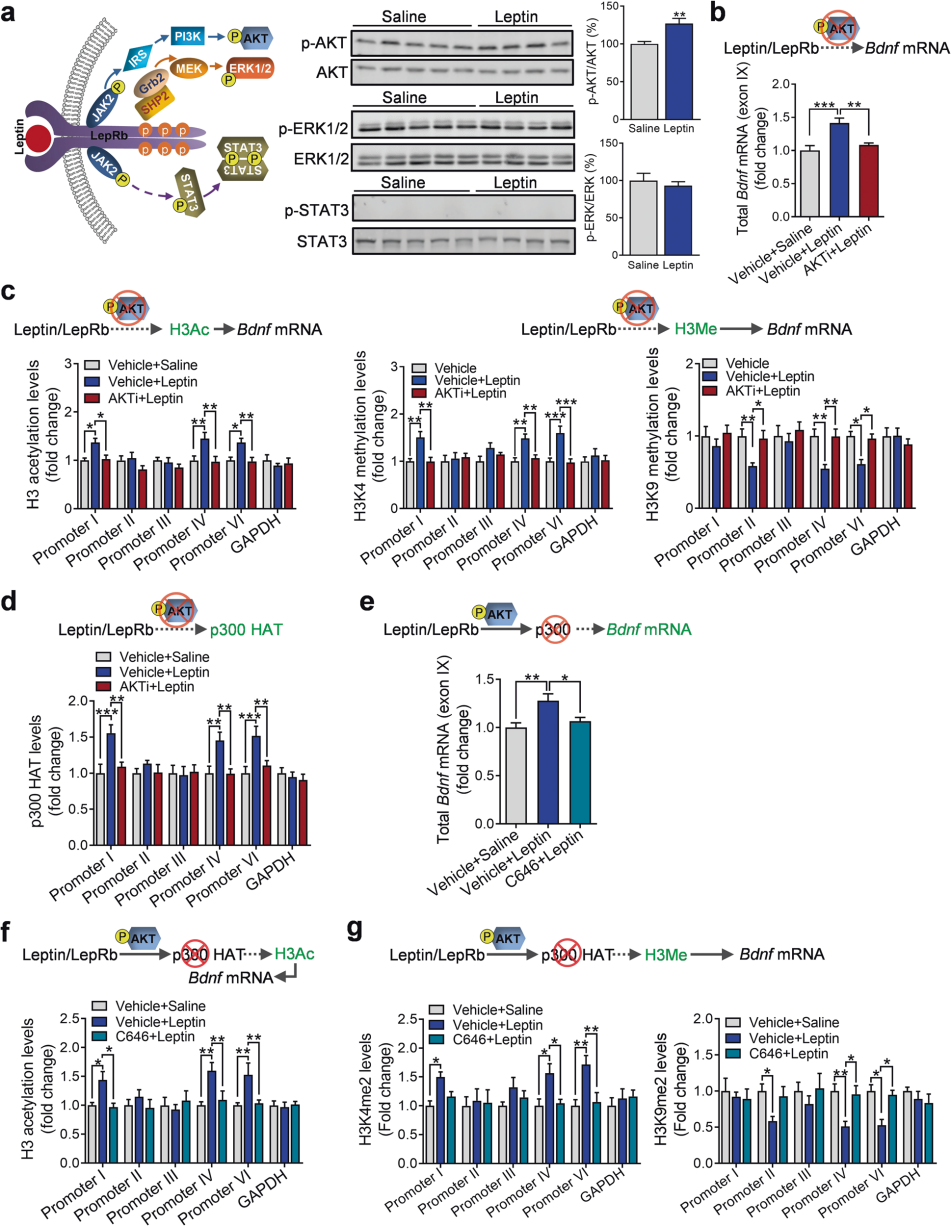
Leptin-induced histone modifications of the *Bdnf* gene are mediated by stimulation of an AKT/p300 HAT cascade. **a** Left, schematic representation of leptin-mediated signaling pathways; middle: western blotting images showing phosphorylation of AKT, ERK, and STAT3 in the hippocampus induced by leptin; right, quantitative data from western blots. Saline, *n* = 5; leptin, *n* = 4. Blockade of leptin-induced increases in total *Bdnf* mRNA expression (**b**) and changes in H3 acetylation, H3K4 methylation, and H3K9 methylation (**c**) and p300 HAT binding by the AKT inhibitor AKTi (**d**). Vehicle-saline, *n* = 9; vehicle + leptin, *n* = 9; AKTi + leptin, *n* = 8. Blockade of leptin-induced increases in total *Bdnf* mRNA expression (**e**), H3 acetylation (**f**), H3K4 and H3K9 methylation (**g**) by the p300 HAT inhibitor C646. *n* = 6 per group. *P < 0.05; **P < 0.01; ***P < 0.001 compared with saline-treated controls.

The binding of leptin to LepRb activates three major signaling pathways, i.e., JAK2/STAT3, PI3K/AKT, and ERK pathways [68] (Fig. 6a). To explore which signaling pathway(s) is responsible for mediating leptin’s effects on *Bdnf* gene expression, we assessed activation of STAT3, AKT, and ERK in the hippocampus by leptin. Leptin treatment at a dose used for gene expression (i.e., 5 mg/kg, i.p.) failed to induce a change in phosphorylation levels of STAT3 or ERK (*t*_(7)_ = 0.5803, *P* = 0.5782), but significantly increased AKT phosphorylation (*t*_(7)_ = 3.8020, *P* = 0.0067), suggesting that leptin action in the hippocampus may be mediated by AKT signaling (Fig. 6a). Indeed, we have previously shown that AKT activation in the hippocampus mediates leptin’s antidepressant-like behavioral effects [14]. To examine whether AKT signaling is required for leptin-induced *Bdnf* gene expression, an AKT selective inhibitor, AKTi, was infused into the dentate gyrus to block AKT signaling prior to leptin injection. We found that inhibition of AKT activation eliminated the effects on total *Bdnf* mRNA expression induced by leptin (Kruskal–Wallis test, *P* < 0.001) (Fig. 6b). In addition, pretreatment with AKTi blocked the effects of leptin on acetylation of H3 at promoters I, IV, and VI and methylation of H3K4 at promoters I, IV, and VI and H3K9 at promoters II, IV, and VI (Fig. 6c) (H3, treatment: *F*_(2,138)_ = 12.1000, *P* < 0.001; promoter: *F*_(5,138)_ = 3.7980, *P* = 0.0030; treatment × promoter interaction: *F*_(10,138)_ = 2.1530, *P* = 0.0242); H3K4, treatment: *F*_(2,138)_ = 20.4300, *P* < 0.001; promoter: *F*_(5,138)_ = 1.2300, *P* = 0.2982; treatment × promoter interaction: *F*_(10,138)_ = 1.9550, *P* = 0.0428; H3K9, treatment: *F*_(2,138)_ = 11.9700, *P* < 0.001; promoter: *F*_(5,138)_ = 1.5530, *P* = 0.1774; treatment × promoter interaction: *F*_(10,138)_ = 1.5140, *P* = 0.1403. These results suggest that the PI3K/AKT pathway plays a critical role in leptin-induced regulation of *Bdnf* gene expression.

AKT can phosphorylate p300 HAT at Ser-1834, thereby increasing its promoter recruitment and histone acetylation [69]. We then examined whether AKT activation is involved in p300 HAT binding activity at *Bdnf* promoters. Leptin was found to increase p300 HAT binding to *Bdnf* promoters I, IV, and VI, and this effect was abolished by pretreatment with AKTi in the hippocampus (treatment: *F*_(2,138)_ = 14.2600, *P* < 0.001; promoter: *F*_(5,138)_ = 4.0820, *P* = 0.0017; treatment × promoter interaction: *F*_(10,138)_ = 2.5300, *P* = 0.0079) (Fig. 6d). Next, we tested whether p300 HAT activity is necessary for leptin-induced changes in *Bdnf* gene expression. C646, a competitive p300 HAT inhibitor [70], was injected into the dentate gyrus prior to leptin treatment. Pretreatment with C646 was sufficient to block leptin-induced increase in total *Bdnf* mRNA expression (*F*_(2,15)_ = 6.9240, *P* = 0.0074) (Fig. 6e) and subsequent acetylation of histone H3 at promoters I, IV, and VI was abolished (treatment: *F*_(2,90)_ = 9.8950, *P* < 0.001; promoter: *F*_(5,90)_ = 2.2800, *P* = 0.0532; treatment × promoter interaction: *F*_(10,90)_ = 2.1570, *P* = 0.0277) (Fig. 6f), suggesting that p300 HAT mediates histone acetylation at *Bdnf* promoters. p300 HAT-mediated histone acetylation events have been shown to modulate H3 methylation [71]. We therefore examined whether methylation of H3K4 and H3K9 at *Bdnf* promoters is affected by p300 HAT activity. Pretreatment with C646 was found to abolish leptin-induced increase in H3K4 methylation and decrease in H3K9 methylation (H3K4, treatment: *F*_(2,90)_ = 12.1900, *P* < 0.001; promoter: *F*_(5,90)_ = 0.9871, *P* = 0.4302; treatment × promoter interaction: *F*_(10,90)_ = 1.1900, *P* = 0.3083; H3K9, treatment: *F*_(2,90)_ = 10.7300, *P* < 0.001; promoter: *F*_(5,90)_ = 0.8255, *P* = 0.5348; treatment × promoter interaction: *F*_(10,90)_ = 1.1440, *P* = 0.3393) (Fig. 6g), suggesting p300 HAT activity is also indirectly involved in histone H3 lysine methylation. Together, these results suggest that leptin induces *Bdnf* gene expression through an AKT/p300 HAT/H3 modification cascade, leading to chromatin remodeling and transcriptional activation.

### *Bdnf* gene expression in the dentate gyrus is essential for the antidepressant-like effect of leptin

**Fig. 7:**
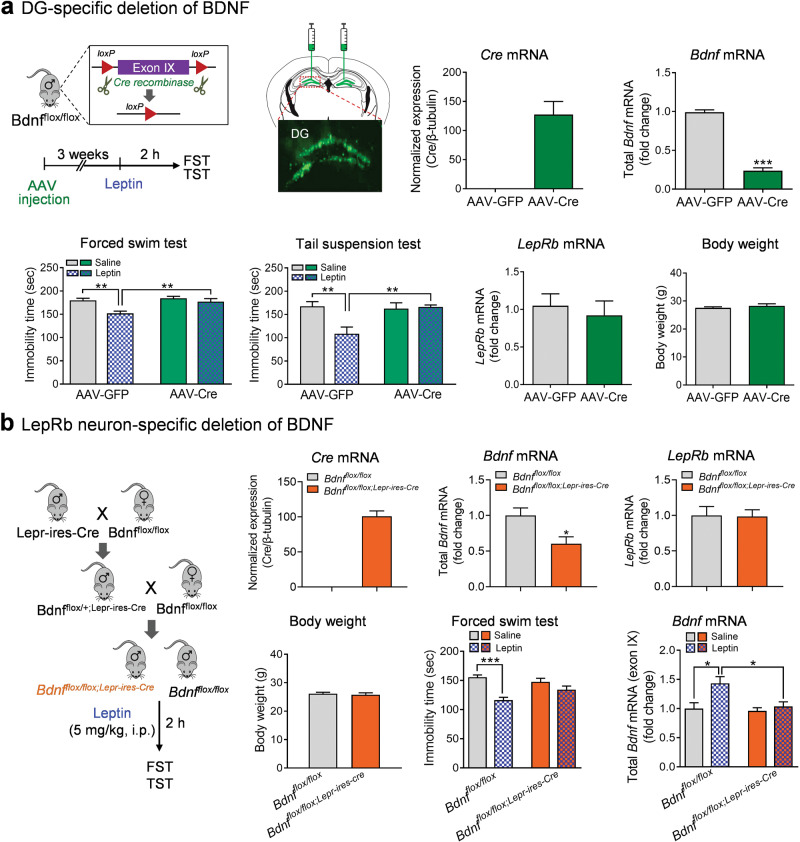
Loss of *Bdnf* in the dentate gyrus abolishes leptin’s antidepressant-like effects. **a** Dentate gyrus (DG)-specific deletion of Bdnf. Upper left, schematic diagram of the Bdnf floxed allele after AAV-Cre-mediated recombination. Upper middle-left, stereotaxic injection of AAV-Cre-GFP or AAV-GFP vectors in the DG of *Bdnf*^*flox/flox*^ mice; upper middle-right, Cre mRNA expression; upper right, total *Bdnf* mRNA expression in the DG. Lower left, forced swim test (FST); lower middle-left, tail suspension test (TST); lower middle-right, LepRb mRNA expression in the DG; lower right, body weight. AAV-GFP + saline, *n* = 10; AAV-GFP + leptin, *n* = 10; AAV-Cre + saline, *n* = 9; AAV-GFP + leptin, *n* = 8. Total AAV-GFP, *n* = 20; total AAV-Cre, *n* = 17. **b** Left panel, schematic diagram of breeding strategy. Upper panel, Cre (left), total *Bdnf* (middle), and LepRb (right) mRNA expression in the DG. *n* = 5 per group. Lower left, forced swim test; *Bdnf*^*flox/flox*^ + saline, *n* = 11, *Bdnf*^*flox/flox*^ + leptin, *n* = 11, *Bdnf*^*flox/flox;Lepr-ires-Cre*^ + saline, *n* = 11, *Bdnf*^*flox/flox;Lepr-ires-Cre*^ + leptin, *n* = 12. Lower middle, body weight; *Bdnf*^*flox/flox*^, *n* = 22, *Bdnf*^*flox/flox;Lepr-ires-Cre*^, *n* = 23. Lower right, total *Bdnf* mRNA level 2 h after leptin injection (5 mg/kg, i.p.). *n* = 5 per group. *P < 0.05; ** *P* < 0.01; *** *P* < 0.001 compared with WT littermates or saline-treated controls.

We next asked whether *Bdnf* gene expression in the dentate gyrus is required for leptin’s depression-related behaviors. To address this question, AAV-Cre and AAV-GFP control vectors were injected into the dentate gyrus of the hippocampus of *Bdnf*^*flox/flox*^ mice (Fig. 7a). Three weeks later, the expression of Cre expression and the deletion of *Bdnf* exon IX in the dentate gyrus were confirmed (*Bdnf* exon IX: Mann–Whitney test, *P* < 0.001) (Fig. 7a). AAV-Cre and AAV-GFP-treated mice received i.p. injection of leptin (1 or 5 mg/kg, i.p.) 2 h before the forced swim and tail suspension tests. Leptin decreased immobility time in both tests in AAV-GFP treated control mice; however, this effect was eliminated in AAV-Cre treated mice (Fig. 7a) (forced swim test, 1 mg/kg, 2 h: treatment: *F*_(1,33)_ = 12.2700, *P* = 0.0013; genotype: *F*_(1,33)_ = 8.7090, *P* = 0.0058; genotype × treatment: *F*_(1,33)_ = 4.1910, *P* = 0.0487; tail suspension test, 5 mg/kg, 2 h: treatment: *F*_(1,33)_ = 5.9060, *P* = 0.0207; genotype: *F*_(1,33)_ = 5.3540, *P* = 0.0270; genotype × treatment: *F*_(1,33)_ = 7.5930, *P* = 0.0095). This insensitivity to leptin is unlikely to be due to a downregulation of LepRb in the dentate gyrus caused by deletion of *Bdnf*, as LepRb mRNA levels were unchanged in AAV-Cre treated mice (Mann–Whitney test, *P* = 0.5567) (Fig. 7a). Moreover, the deletion of *Bdnf* in the dentate gyrus had no effect on body weight (*t*_(35)_ = 0.8378, *P* = 0.4078) (Fig. 7a).

Given that LepRb neurons expressed *Bdnf* in the dentate gyrus, we asked whether an intracellular interaction between LepRb and *Bdnf* mediates leptin action on despair behavior. To address this, Lepr-ires-Cre mice were crossed with *Bdnf*^*flox/flox*^ mice to induce deletion of *Bdnf* specifically in LepRb neurons (Fig. 7b). The resultant *Bdnf*^*flox/flox;Lepr-ires-Cre*^ mice confirmed Cre expression in the dentate gyrus with a dramatic reduction of *Bdnf* expression (*t*_(8)_ = 2.7830, *P* = 0.0238) but normal levels of LepRb mRNA (*t*_(8)_ = 0.0925, *P* = 0.9286) (Fig. 7b). *Bdnf* ^*flox/flox;Lepr-ires-Cre*^ mice and *Bdnf*^*flox/flox*^ littermate controls were injected with leptin (5 mg/kg, i.p.) 2 h before the forced swim test. We found that the targeted deletion of *Bdnf* in LepRb neurons abolished leptin’s antidepressant-like effects in *Bdnf*^*flox/flox;Lepr-ires-Cre*^ mice (treatment: *F*_(1,41)_ = 23.5300, *P* < 0.001; genotype: *F*_(1,41)_ = 0.8602, *P* = 0.3591; genotype × treatment: *F*_(1,41)_ = 5.7670, *P* = 0.0210) (Fig. 7b). As seen in mice with the deletion of *Bdnf* in the dentate gyrus, body weight was unaltered in *Bdnf* ^*flox/flox,Lepr-ires-cre*^ mice (*t*_(43)_ = 0.4250, *P* = 0.6729) (Fig. 7b). Lastly, we tested whether leptin-induced *Bdnf* gene expression is dependent on *Bdnf* expression in LepRb neurons. Total *Bdnf* mRNA levels were measured 2 h after leptin treatment (5 mg/kg, i.p.). We showed that leptin-induced increase in total *Bdnf* mRNA expression was eliminated by deletion of *Bdnf* in LepRb neurons (treatment: *F*_(1,16)_ = 8.0840, *P* = 0.0117; genotype: *F*_(1,16)_ = 5.8620, *P* = 0.0277; genotype × treatment: *F*_(1,16)_ = 3.9460, *P* = 0.0644) (Fig. 7b), suggesting that leptin regulates *Bdnf* gene expression via a direct mechanism.

## Discussion

The hippocampus is a highly vulnerable, dynamic, and responsive brain structure in that it demonstrates rapid plasticity at the molecular, cellular, circuit, and functional levels [72, 73]. BDNF, which is expressed at the highest levels in the hippocampus, plays a vital role in hippocampal plasticity and function [74, 75]. In this study, our finding that dentate gyrus neurons expressing the functional leptin receptor isoform LepRb also contained BDNF suggests that BDNF-expressing neurons are targets of leptin action. Further studies showed that leptin upregulated total *Bdnf* gene expression and protein synthesis, causing activation of TrkB in the hippocampus. *Bdnf* transcription is uniquely controlled by nine individual promoters, which drive expression of multiple transcripts encoding the same protein [34–36]. Such complex gene structure suggests that *Bdnf* gene expression is finely tuned. We found that leptin treatment increased transcriptional activity of *Bdnf* promoters I, IV, and VI in the hippocampus. However, leptin deficiency or loss of its receptor decreased *Bdnf* gene expression, albeit via suppression of transcriptional activity at different promoters. Furthermore, leptin activated AKT signaling, which in turn regulated p300 HAT recruitment and subsequently promoted the transcriptional activity of *Bdnf* promoters via an epigenetic mechanism. Importantly, the loss of *Bdnf* gene expression in the dentate gyrus eliminated the antidepressant-like behavioral effects of leptin, suggesting that upregulation of BDNF underlies leptin’s action on mood-related behavior.

BDNF in the hippocampus has been implicated in the pathophysiology of depression and the therapeutic mechanisms of antidepressant treatments [23, 47, 76]. In animal studies, BDNF levels in the hippocampus decrease in response to a variety of stressors and increase following various antidepressant treatments [76]. Postmortem studies demonstrate that levels of BDNF in the hippocampus (both mRNA and protein) are reduced in depressed patients [77, 78], whereas BDNF levels are elevated in patients receiving antidepressants [26, 77–80]. Moreover, overexpressing BDNF in excitatory neurons of the forebrain (including hippocampus) caused an antidepressant-like behavioral phenotype [81], while a single infusion of BDNF directly into the hippocampus was sufficient to induce an antidepressant-like effect [29]. Importantly, loss of forebrain BDNF or reduced BDNF signaling (overexpression of truncated TrkB) attenuates the antidepressant-like response to antidepressants [27, 82–84]. The rapid acting antidepressant ketamine is also dependent on increased BDNF signaling [30, 85]. Furthermore, mice with reduced BDNF signaling caused by overexpressing truncated TrkB are resistant to the effects of antidepressants [27]. We have shown that leptin has antidepressant-like effects mediated by LepRb in the hippocampus [13–15, 22, 53]. Our findings that leptin increased *Bdnf* gene expression, while the loss of BDNF abolished leptin’s antidepressant-like effects, support the idea that hippocampal BDNF is required for leptin action on mood-related behavior. Furthermore, this effect is likely to be mediated by direct interactions between LepRb and BDNF, as deletion of *Bdnf* from LepRb neurons in the dentate gyrus eliminated the antidepressant-like effects of leptin.

*Bdnf* gene expression is regulated by diverse stimuli and physiological and pathological conditions through differential recruitment of individual exon-specific *Bdnf* promoters. *Bdnf* transcripts containing exons I, II, III, IV, and VI are present in the hippocampus, and they make differential contributions to total BDNF [86]. Many antidepressants have been shown to result in distinct exon-specific *Bdnf* transcripts [44, 87–89]. For example, tranylcypromine and desipramine increase exon I, II, and IV (original III) mRNAs, fluoxetine treatment enhances activity of promoter II [88], whereas reboxetine increases activation of promoter IV [87]. Leptin, similar to classic antidepressants, increased total *Bdnf* gene expression while inducing sexually dimorphic profiles of *Bdnf* promoter activation, with male mice exhibiting increased expression of exons I, IV, and VI and female mice exhibiting increased expression of exons I, II, III, and VI. In contrast, leptin deficiency or leptin receptor deficiency decreased total *Bdnf* gene expression. However, three distinct lines of male mice deficient in leptin or leptin signaling, i.e., *ob/ob*, *db/db* and *Lepr*^*flox/flox;Emx1-Cre*^ mice, exhibited different patterns of downregulation of exon-specific *Bdnf* gene expression, with *ob/ob* associated with decreased expression of exons II, III, IV, and VI, *db/db* associated with decreased expression of exons I, III, and IV and Lepr^*flox/flox;Emx1-Cre*^ associated with decreased expression of exons I, II, IV, and VI. In *ob/ob* mice, replenishing leptin restored the levels of transcriptional activity at promoters IV and VI but failed to reverse the downregulation of exons II and III. These results suggest that leptin deficiency is responsible for the downregulation of exons IV and VI expression, but not exons II and III. We considered the possibility that the downregulated expression of exons II and III in *ob/ob* mice was caused by obesity, which cannot be normalized by acute leptin treatment. This seems to be the case for exon III but not for exon II, as expression of this exon was also decreased in *Lepr*^*flox/flox;Emx1-Cre*^ mice with normal body weight. *Bdnf* regulation appears to be even more complicated in female mice. First, leptin increases the transcription of the *Bdnf* gene in proestrus but not diestrus, with specific changes in expression of exons I, II, III, and VI. Second, while leptin deficiency caused a reduction of expression of exons I, III, and VI in female *ob/ob* mice, female *db/db* mice exhibited decreased expression levels of exons I, III, IV, and VI. *Bdnf* promoters show differential transcription efficiency with promoters IV and VI contributing more substantially to total BDNF in the hippocampus [86]. How the different patterns of *Bdnf* exon-specific upregulation or downregulation induced by leptin, leptin deficiency and leptin receptor deficiency contribute to total BDNF are dependent on the relative abundance of individual *Bdnf* transcripts and the fold changes of their relative abundance following different manipulations. Our results demonstrate that transcription of the *Bdnf* gene is tightly regulated by leptin, displaying distinct exon-specific, sex-dependent expression profiles in response to elevated leptin levels, defective leptin signaling, and leptin resistance. These findings suggest that *Bdnf* expression in the hippocampus is sensitive to fluctuations in leptin availability and leptin signaling, serving as a finely tuned neural component of the adipose-brain axis.

**Fig. 8:**
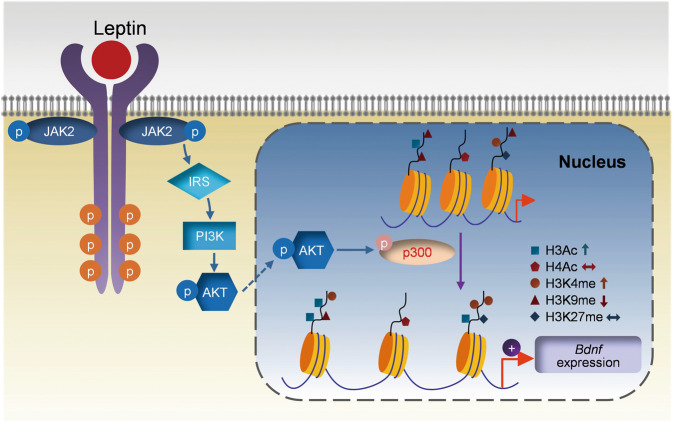
Schematic diagram illustrating the mechanism by which leptin induces epigenetic regulation of *Bdnf* gene transcription. Leptin binds to the long leptin receptor isoform, LepRb, and activates AKT signaling, which in turn phosphorylates p300 HAT, resulting in histone modifications at *Bdnf* exon-specific promoters and thereby promoting chromatin remodeling and gene transcription.

One intriguing and important finding of this study is that leptin activates the PI3K/AKT pathway to recruit the p300 HAT, leading to histone modifications and subsequently transcriptional activation of the *Bdnf* gene (Fig. 8). This finding constitutes strong evidence for an intersection between cell signaling and epigenetic regulation. First, among three major signaling pathways downstream of LepRb, the AKT pathway was selectively activated in the hippocampus by leptin. Moreover, we have previously shown that AKT signaling in the hippocampus is required for leptin’s antidepressant-like behavioral effects [14]. Second, AKT can phosphorylate p300 HAT at Ser-1834 [69], which could induce its recruitment to the *Bdnf* promoter, leading to the acetylation of histones and activation of the transcriptional machinery. Indeed, leptin treatment increased p300 HAT binding to *Bdnf* promoters I, IV, and VI, and this effect was attenuated by inhibition of AKT. Furthermore, C646, a p300 HAT inhibitor, attenuated leptin-induced acetylation of histone H3. AKT can also phosphorylate other chromatin-modifying enzymes, such as the histone methyltransferase EZH2 (H3K27 methylation) and DNA methyltransferase DNMT1, resulting in changes in function of these enzymes [90, 91]. Unlike histone acetylation, which occurs rapidly in response to stimuli, histone methylation, and DNA methylation are relatively static [90]. In this study, we analyzed the methylation level of 76 CpG sites (6 CpG islands) within the promoters I, II, IV, and VI and the exonic regions following leptin treatment. The methylation at 75 CpG sites was unaltered by acute leptin treatment, suggesting that DNA methylation may not play a significant role in leptin-induced *Bdnf* gene expression. However, we cannot rule out the possibility that other CpG sites we did not analyze may be sensitive to leptin treatment. Methylation of histones at different residues results in different transcriptional outcomes. For example, methylation of H3K27 and H3K9 is associated with transcriptional repression, whereas methylation on H3K4 usually increases gene expression [92–94]. We observed increased H3K4 methylation coupled with decreased H3K9 methylation with no changes in H3K27 methylation at the *Bdnf* promoter following leptin treatment. Interestingly, blockade of p300 HAT with C646 also abolished leptin-induced changes in methylation of H3K4 and H3K9. The involvement of p300 HAT in the regulation of histone H3 methylation at the *Bdnf* promoter may be secondary to its effect on histone H3 acetylation. In support of this, one study reported that H3K4 methylation is dependent upon p300 HAT-mediated H3 acetylation [71]. The methyltransferase SET1C, which methylates histone H3 at lysine 4, was demonstrated to act synergistically with p300 HAT through direct interactions and coupled histone modifications [71]. Our results support that direct communications between the AKT signaling pathway and the chromatin-modifying machinery mediates *Bdnf* gene expression in response to leptin. This may represent a generalized epigenetic mechanism that permits cells to respond dynamically to environmental signals.

Our findings support the idea that leptin interacts with BDNF via LepRb in the hippocampus to exert its behavioral effects, beyond its role as an adiposity signal. Although the present study only investigated leptin’s antidepressant-like effects, the interactions between leptin signaling and BDNF hypothetically could extend to other hippocampal functions, such as learning and memory [18, 95, 96]. Moreover, regulation of *Bdnf* gene expression by leptin signaling is complex, with differential regulation of exon-specific expression in a sex-dependent manner. BDNF, in turn, is required for leptin action in the hippocampus. Thus, dysregulation of BDNF presumably could lead to resistance to leptin, contributing to the pathogenesis of depression and Alzheimer’s disease.
